# On the concept, taxonomy, and transculturality of disordered grief

**DOI:** 10.3389/fpsyg.2023.1165421

**Published:** 2024-02-05

**Authors:** Afonso Gouveia

**Affiliations:** ^1^Psychiatry Service, Department of Mental Health, Local Health Unit of Baixo Alentejo, Beja, Portugal; ^2^CHRC, NOVA Medical School, Universidade Nova de Lisboa, Lisbon, Portugal

**Keywords:** grief, bereavement, prolonged grief, transcultural, pathological grief, taxonomy, diagnosis, culture

## Abstract

The enduring question of whether grief can ever be pathological (and, if so, when) has been shrouding mental health and psychiatric care over the last few years. While this discussion extends beyond the confines of psychiatry to encompass contributions from diverse disciplines such as Anthropology, Sociology, and Philosophy, scrutiny has been mainly directed toward psychiatry for its purported inclination to pathologize grief—an unavoidable facet of the human experience. This critique has gained particular salience considering the formal inclusion of prolonged grief disorder (PGD) in the 11th edition of the International Classification of Diseases (ICD-11) and the subsequent Text Revision 5th Edition of the Diagnostic and Statistical Manual of Mental Disorders (DSM-5-TR). This study contends that the inclusion of prolonged grief disorder as a diagnostic entity may be excessively rooted in Western cultural perspectives and empirical data, neglecting the nuanced variations in the expression and interpretation of grief across different cultural contexts. The formalization of this disorder not only raises questions about its universality and validity but also poses challenges to transcultural psychiatry, due to poor representation in empirical research and increased risk of misdiagnosis. Additionally, it exacerbates the ongoing concerns related to normativism and the lack of genuine cultural relativism within the DSM. Furthermore, the passionate discussion surrounding the existence, or not, of disordered forms of grief may actually impede effective care for individuals genuinely grappling with pathological forms of grief. In light of these considerations, this study proposes that prolonged grief disorder should be approached as a diagnostic category with potential Western cultural bias until comprehensive cross-cultural studies, conducted in diverse settings, can either substantiate or refute its broader applicability. This recalibration is imperative for advancing a more inclusive and culturally sensitive understanding of grief within the field of psychiatry.

## 1 Introduction

Grief, bereavement, and mourning are terms that are somewhat interchangeably used among laypersons. That is not to say that they are the same, just that they have some meanings in common. Conceptually, ‘grief’ encompasses the set of diverse reactions (psychological, physical, behavioral, social…) that follow losing someone, or something, that is closely related to the individual. It constitutes a normal, expectable process of life, and though it is usually associated with following death, it can also arise from a social loss (e.g., relationship or job loss). Bereavement, on the other hand, corresponds to the period of mourning and grief, i.e., after the loss, and its duration depends on several factors (circumstances of the loss, level of attachment, etc.). Finally, mourning can be conceptualized as the culturally influenced process by which people adapt to a life without that someone or something (Casarett et al., 2001, p. 213, 214; Lally et al., 2019, p. 10,796).

Following the 11th edition of the International Classification of Diseases (ICD-11) and the recent Text Revision of the 5th Edition of the Diagnostic and Statistical Manual of Mental Disorders (DSM-5-TR), the term grief will be favored. Similarly, so as not to mix the general concept of abnormal grief with more specific grief-related concepts, the phrase *disordered grief* will also be favored when not referring to a particular construct.

The present study sets out from an exploratory standpoint, aiming to address the nosology of disordered grief and its problematics. In the second part, the current concept of disordered grief will be addressed from a taxonomic standpoint, before concluding with a transcultural shortcoming of the actual prolonged grief disorder (PGD) in the DSM-5-TR. Aiming for a more interesting and useful debate on how to study grief-related disorders in a multi-cultural, ever-changing, globalized world, I challenge PGD’s non-Western validity. Rather than finish the job, I aim to sketch some potentially fruitful themes to explore.

## 2 Subsections relevant to the subject

### 2.1 A brief note on the natural history of grief

#### 2.1.1 Why do we grieve? An evolutionary take by Horwitz and Wakefield

“As long as emotions have been recorded, experiences of grief have been central portrayals of basic human nature” (Horwitz and Wakefield, 2007, p. 30).

Several reasons have been appointed to justify how sadness is a normal, biologically designed response. Among them, we can find evidence that supports the continuity of sadness across species and cultures, as well as the presence of loss responses even in pre-socialized infants (Horwitz and Wakefield, 2007, p. 38–41). Studies of primates and very young children, as well as cross-cultural studies, indicate that sadness responses are biological and not merely socially learned. Human beings appear to have some natural and biological design to develop sadness in certain situations, especially when losses are involved. That being said, this biological determinism does not preclude social influences on the expression of sadness, particularly concerning the *how* and *when*. Normal sadness can be said to be produced by a conjunction of biology and culture (Horwitz and Wakefield, 2007, p. 42).

Regarding the question of what survival advantage could have an emotion such as sadness conferred to be naturally selected, given its painful and, sometimes, debilitating qualities, Horwitz and Wakefield hold that, even though the function of sadness remains a largely unanswered and debated subject, some plausible hypotheses about the functions of sadness have been drawn, such as (1) increasing fitness precisely by making people less active and motivated; (2) communicating inner states to other people and attracting social support after losses (which seems particularly plausible in grief); (3) allowing social bonds to persist during temporary absences of a significant other; (4) protecting from aggression after status losses, i.e., by signaling acceptance of defeat; and (5) disengaging people from unproductive efforts, inviting instead the person to re-evaluate their future (Horwitz and Wakefield, 2007, p. 47–50). Even though these are far from established, the sheer existence of plausible reasons for sadness to have some sort of adaptive function reinforces the idea that sadness, when proportionate, constitutes an aspect of human nature that occurs by design (Horwitz and Wakefield, 2007, p. 51).

For an extended version of this account, refer to the original study by Horwitz and Wakefield (2007).

#### 2.1.2 Culture and grief

The disposition to experience strong sadness in response to loss events appears to be universal in all humans (Horwitz and Wakefield, 2007, p. 41). The psychologist Paul Ekman tried to test the universality of emotions, including sadness, by studying facial expressions across individuals from several cultures, including the Fore culture in Papua, New Guinea, which was free of contact with outside cultures. Ekman sought to study emotions through facial expressions because, contrary to verbal reports, they are less prone to cultural influences. Indeed, Ekman’s findings supported the thesis that expressions of sadness are universally present in all cultures, with some innate and evolutionarily selected features. Moreover, culture and biological design are sometimes complementary, as is the case with emotions. Culture itself is an evolved human capacity, and humans are designed to be capable of socializing and integrating social rules, meanings, and values. Cultural meanings appear to play an essential, and maybe even designed, role in forming the final emotional expression. Emotionality is, thus, biologically selected to be capable of cultural variation (Horwitz and Wakefield, 2007, p. 42, 43).

Moreover, the categories that trigger sadness are common across all societies. First, while cultural meanings influence which events are regarded as losses, contextual factors, such as humiliation or entrapment, determine the severity of the loss. In other words, as Horwitz and Wakefield summarized, “nature supplies the template for triggers of loss responses, but culture provides the content for this template” (Horwitz and Wakefield, 2007, p. 43, 44). Second, cultural values are directly involved in setting the duration and intensity of a loss response; in other words, cultural values play a part in shaping how much emotion and which aspects of the loss response people ought to express and show in public (Horwitz and Wakefield, 2007, p. 44).

Overt emotional expression is guided by cultural norms, or “scripts,” that guide the overt expression of emotion. While many non-Western cultures encourage the expression of sadness in public ceremonies and organized rituals, others strongly discourage demonstrations of extreme sadness, restricting outward grief to just 4 days, after which the bereaved person is not expected to show grief or refer to the deceased, such as in the Navaho culture. The Kaluli tribe of New Guinea responds to loss with outward anger that is turned into feelings of being owed compensation, as opposed to developing self-blame or guilt over the loss. Through public ceremonies, widows/widowers allow the expression of their feelings through weeping, songs, and the payment of compensation. Meanwhile, Mediterranean societies developed longer periods of mourning for bereaved widows, which could last for many years (Horwitz and Wakefield, 2007, p. 44).

The cultural norms for expressing emotions, however, should not be mistaken for the actual experienced emotions. In Iranian culture, if some relative passes away, the bereaved must really act like they are sorry; otherwise, he or she might be accused of ill feelings toward the deceased, in particular, if the person stands to inherit something. Cultural norms, on the other hand, can even turn grief expressions into cheerfulness, such as in the Irish wake. Another example would be Balinese’s response to bereavement with laughter. However, even when cultural norms determine expressive responses seemingly incompatible with sadness, they recognize, nonetheless, that the distinctive underlying feeling is sadness. As such, even though the Balinese believe that sadness is the natural response to loss, they also believe that its expression should be countered because it leads others to be sad and is seen as detrimental to health. Chinese people, even though feeling intense sadness, tend to concentrate on bodily feelings of distress (back pain, stomach aches, headaches, etc.). Despite being aware of the psychological aspects influencing their feelings, social norms mandate them to express their problems in somatic terms. All in all, despite the different outward manifestations, common underlying emotions appear to be universal (Horwitz and Wakefield, 2007, p. 44, 45).

Strong interpersonal ties, networks of social support, powerful collective religious rituals, and belief systems help to make people less vulnerable to loss. For instance, while the Kaluli have ritualized group ceremonies after the loss, other societies mandate the replacement of the deceased spouse with a new one, abbreviating the grief response (Horwitz and Wakefield, 2007, p. 45, 46).

Given that culture influences the proportionality of the sadness response, it can be argued that no objective transcultural biological threshold, between normal and dysfunctional grief, exists. In fact, because the very same response may be regarded as normal in one culture, but disordered in another, grief can be said to imply cultural relativity. The reason is not that cultures directly, or arbitrarily, define normal vs. disordered sadness responses, but rather that different thresholds exist across different cultures, since they induce different intensities and durations of response to specific causes of sadness. Such varying meanings must surely be considered when judging whether a bereaved person is responding normally or not. Cultural norms are, thus, a significant and undeniable part of the grief response. Naturally, then, cultural norms cannot be overlooked when trying to ascertain whether a grief response is best explained in terms of design or dysfunction (Horwitz and Wakefield, 2007, p. 46).

### 2.2 Conception and taxonomy of disordered forms of grief

#### 2.2.1 The concept of disordered grief

The conceptual history of pathological forms of grief has been a convoluted one. While this history would merit an entire study of its own (perhaps even a book), it matters to say how grief has been under a long discussion on whether it can become disordered and, if so, starting when—how long is *too long*? How intense is *too intense*? How crippling is *too crippling*? These questions, among others, have not been answered to a reasonably consensual degree, and they have set the stage for a public outcry somewhat reminiscent of antipsychiatry movements. Those skeptics claim psychiatry is pathologizing/medicalizing grief, reducing the dignity of the survivor, implying that survivors should get over their loss faster, and fashioning pathology out of love. Additionally, they claim bereaved people feel stigmatized, or even outright offended, by having their distress labeled a mental disorder. This public uproar, while valuable to a degree, can sometimes obfuscate the issue in an unproductive manner, frequently focusing on clear-cut cases, accomplishing very little besides further entrenching parties, and relinquishing a much more needed discussion on the borderland and on culture relativism.

A number of concepts involving disordered grief can be found in the literature. Pathological grief, complicated grief, or complex bereavement, leading to nowadays’ prolonged grief, are some of them, but several other grief-related concepts have also been formulated, such as delayed, anticipatory, hypertrophic, chronic, or traumatic grief (Boland et al., 2022, p. 850, 851), illustrating how grief is a rather dynamic subject.

Both ICD-10 and DSM-IV did not partake in distinguishing normal grief from disordered grief [World Health Organization (WHO), 1993; American Psychiatric Association (APA), 1994]. However, after considering empirical evidence extending as early as 1995 (Prigerson et al., 1995), a diagnostic set of criteria for PGD was eventually proposed for inclusion (Prigerson et al., 2009; Boelen and Prigerson, 2012). Both the World Health Organization (WHO) and the American Psychiatric Association (APA) ended up featuring PGD in their ICD-11 and DSM-5-TR, respectively. The diagnostic criteria were reportedly set after a statistical analysis of the proposed criteria, as agreed, and developed, by a panel of experts (Prigerson et al., 1999, 2009), consisting of the most accurate markers of bereaved persons suffering from painful, destructive, persistent forms of grief (Prigerson et al., 2009).

PGD is described as an intense yearning and/or persistent preoccupation for the deceased. It involves thoughts of the deceased after his/her death, along with other grief-related symptoms (emotional numbness, intense emotional pain, and avoidance of reminders that the person is deceased), extending for over at least 6 months (ICD-11) or 12 months (DSM-5-TR). In children and adolescents, this preoccupation may focus on the circumstances of the death. Finally, PGD needs to be sufficiently severe to cause impairment in functioning [First et al., 2022, p. 218; World Health Organization (WHO), 2022; American Psychiatric Association (APA), 2022a,b].

Below, in Table 1, the PGD criteria are listed according to DSM-5-TR and ICD-11.

**Table 1:** Criteria and current conception of disordered grief according to diagnostic manuals.

World Health Organization (WHO), (2022)6B42 Prolonged grief disorder	Prolonged grief disorder is a disturbance in which, following the death of a partner, parent, child, or other person close to the bereaved, there is persistent and pervasive grief response characterized by longing for the deceased or persistent preoccupation with the deceased accompanied by intense emotional pain (e.g., sadness, guilt, anger, denial, blame, difficulty accepting the death, feeling one has lost a part of one’s self, an inability to experience positive mood, emotional numbness, and difficulty in engaging with social or other activities). The grief response has persisted for an atypically long period of time following the loss (more than 6 months at a minimum) and clearly exceeds expected social, cultural, or religious norms for the individual’s culture and context. Grief reactions that have persisted for longer periods that are within a normative period of grieving given the person’s cultural and religious context are viewed as normal bereavement responses and are not assigned a diagnosis. The disturbance causes significant impairment in personal, family, social, educational, occupational, or other important areas of functioning. (World Health Organization (WHO), 2022)
American Psychiatric Association (APA) (2022a)F43.8Prolonged Grief Disorder (Trauma and Stressor-Related Disorders)	Prolonged grief disorder represents a prolonged maladaptive grief reaction that can be diagnosed only after at least 12 months (6 months in children and adolescents) have elapsed since the death of someone with whom the bereaved had a close relationship (Criterion A). Although in general this time frame reliably discriminates normal grief from grief that continues to be severe and impairing, the duration of adaptive grief may vary individually and cross-culturally. The condition involves the development of a persistent grief response characterized by intense yearning or longing for the deceased person (often with intense sorrow and frequent crying) or preoccupation with thoughts or memories of the deceased, although in children and adolescents, this preoccupation may focus on the circumstances of the death. The intense yearning/longing or preoccupation has been present most days to a clinically significant degree and has occurred nearly every day for at least the last month (Criterion B). Moreover, since the death, at least three additional symptoms have been present most days to a clinically significant degree and have occurred nearly every day for at least the past month. These symptoms include identity disruption since the death (e.g., feeling as though part of oneself has died; Criterion C1); a marked sense of disbelief about the death (Criterion C2); avoidance of reminders that the person is dead, which in children and adolescents may be characterized by efforts to avoid reminders (Criterion C3); intense emotional pain (e.g., anger, bitterness, and guilt) since the death (Criterion C4); having difficulty reintegrating into personal relationships and activities since the death (e.g., problems engaging with friends, pursuing interests, or planning for the future; Criterion C5); emotional numbness (absence or marked reduction of emotional experience) as a result of the death (Criterion C6); feeling that life is meaningless as a result of the death (Criterion C7); or intense loneliness as a consequence of the death (Criterion C8). The symptoms of prolonged grief disorder must result in clinically significant distress or impairment in social, occupational, or other important areas of functioning in the bereaved individual (Criterion D). The nature, duration, and severity of the bereavement reaction must clearly exceed expected social, cultural, or religious norms for the individual’s culture and context (Criterion E). Although there are variations in how grief can manifest, the symptoms of prolonged grief disorder occur across genders and in diverse social and cultural groups. [American Psychiatric Association (APA), 2022a,b, p. 323, 324]

#### 2.2.2 Grief and/or/not depression

Grief may present with a clinical picture very similar to that of depression. They may share symptoms and even co-occur, but there are several important and useful distinctions that can be made at a phenomenological level. Additionally, both depression and grief can present quite heterogeneously on their own. Even though an in-depth differentiation of these experiences lies outside the scope of this study, some of those differences are worth mentioning.

Grief is complex and fluid at the same time, where positive emotions take place in addition to more negative ones, changing and evolving over time, until it gradually lessens and gives room to more positive, comforting aspects of the lost relationship. States of grief usually fluctuate and suffer paroxysms, or pangs of grief, usually related to reminders of the deceased. Grief involves adjustments at a cognitive and behavioral level, which take place, progressively, until the lost person, or thing, is held by the bereaved person in a comfortable place in memory, and he or she is able to resume a normal, fulfilling life (Boland et al., 2022, p. 852). According to Prigerson, “intense, persistent yearning for the deceased person is specifically a characteristic symptom of prolonged grief [PG] but is not a symptom of MDD (or any other DSM disorder)” (Frances, 2012). As noted by Lamb et al. (2010, p. 23), two features that distinguish grief are the remaining sense of connectedness to others, and the sense that things will, or can, get better.

Depression, on the other hand, is more pervasive and impairs self-validating, positive feelings. It usually involves debilitating symptoms accompanied by persistent low mood, tending to be associated with poor work and social functioning. Unless treated, depression also carries neurobiological and other physical changes (Boland et al., 2022, p. 852). Although depressive features, such as lethargy and bodily discomfort, can occur in grief, a pervasive sense of isolation, stasis (or lack of dynamism), and loss of possibilities are useful features to distinguish depression from ‘typical grief’ (Ratcliffe, 2019, p. 541). For an in-depth description of key differences, see Ratcliffe (2019, p. 538–551).

When DSM-5 was released, many psychiatrists were critical of the drop of the bereavement exclusion clause. This decision was maintained in the DSM-5-TR, and, currently, it is technically possible to be depressed and have a PGD diagnosis on top. While this article is not on depression, one has to wonder whether this line of thinking will further complicate things at a time when psychiatric taxonomy faces incredible reliability and validity challenges.

## 3 Discussion

### 3.1 Prolonged grief disorder and *WEIRD* cultural syndromes

#### 3.1.1 On transcultural psychiatry

Unlike physical illnesses, mental illnesses may not be constitutionally universal: “The nature and the possibilities of illness themselves may vary from culture to culture. And hence, there may be genuine, but merely local, illnesses” (Thornton, 2022, p. 53).

DSM-IV’s culture-bound syndromes are “locality-specific patterns of aberrant behavior and troubling experiences that may or may not be linked to a particular DSM-IV diagnostic category” [American Psychiatric Association (APA), 2000, p. 898]. Currently, a derivation of culture-bound syndromes can still be found within the DSM, reflecting DSM’s increasing global use, but also cultural diversity in America. DSM-5 considers three ways on how culture might affect diagnosis and prognosis: (1) cultural syndromes, (2) cultural idioms of distress, and (3) cultural explanations (or perceived causes) of illnesses (or symptoms). Culture syndromes are no more than a cluster of symptoms that (a) co-occur, (b) are relatively unchanging, and (c) are found in a specific culture [American Psychiatric Association (APA), 2013, p. 14, 15]. DSM-5 also describes nine common cultural syndromes in its ‘Glossary of Cultural Concepts of Distress’ [American Psychiatric Association (APA), 2013, p. 833–837]. In those cultural syndromes, the framework of beliefs that surround the concept, and how it differs notoriously from biomedical psychiatry, is of prominence. The cultural syndrome’s illness status is supported by DSM’s linkage of other main diagnostics and categories, rendering it as a local but genuine mental illness (Thornton, 2022, p. 56).

Whether it is possible to aim for reliability, validity, and cultural variation at the same time is a question that is still very much unsettled and debated at the heart of Psychiatric Nosology and Taxonomy.

#### 3.1.2 *WEIRD* cultural syndromes and grief

In 2010, Canadian Researcher Joseph Henrich and colleagues exposed how the world’s top journal empirical studies were largely based on W.E.I.R.D. subjects, that is, *Western*, *Educated*, *Industrialized*, *Rich*, and *Democratic*. Their point was sharp: Behavioral sciences’ databases are drawn from a very thin fraction of humankind. Thus, to what extent can we really generalize findings from such a restricted sample? Indeed, they conclude that *WEIRD* subjects may just be one of the worst populations to generalize from, recommending instead a less cavalier stance in addressing human nature when based on findings drawn from the exceptionally unrepresentative part of humanity that are WEIRD subjects. Henrich and colleagues call on behavioral scientists to stop routinely assuming that their findings are generalizable to the whole species (Henrich et al., 2010, p. 63, 78, 79).

Following this line of thought, Dominic Murphy contends that people in Western countries have their own values and minds, which are not necessarily, nor always, such as those of the rest of humankind, that is not to say that the rest of humanity is all the same, just that Westerners are distinctive in several ways. While these differences are very much real, only now are we beginning to explore the implications they carry for the sciences of the mind and brain. Murphy urges that “WEIRD people might be an exception to the rule, or that there may be no rule at all” (Murphy, 2015, p. 97, 98).

Criticizing the APA, Murphy draws attention to the inconsistency that “only DSM categories can be cross-cultural. (…) American mental illness is universal, and other cultures have specific conditions. The American (indeed, the American of Non-Hispanic European descent) has humanity’s factory model psychology, and other cultures just provide noisy local variation” (Murphy, 2015, p. 100). He then adds the following:

“[C]ulture-bound syndromes are not seen as unreal or artefactual, just not standard. The assumption in mainstream psychiatry is that Western conditions are not culture-bound; they represent abnormalities in a universal human endowment. (…) [DSM-5] treats muscle dysmorphia as a regular diagnosis, not a culture-bound syndrome. This is despite prevalence data for the wider condition of body dysmorphic disorder being noted only for Germany and the USA, and the characteristic behaviours being weightlifting and steroid use, which largely exist in specific western contexts (…) The point is that DSM-5 has main text for diagnoses that occur in the West and a special appendix for the rest [of] the world. It is hard not to see this as the expression of a view that Western minds are the norm. (…) Even if we anticipated that DSM would be used in just a few western countries, the presence of immigrants from other cultures in those countries would raise questions about whether the manual was handling cross-cultural issues adroitly” (Murphy, p. 100, 101).

Despite acknowledging that DSM-5 has committed to a more varied picture of mental illness, Murphy warns that these DSM diagnostic categories are infiltrating developing countries and non-Western cultures as more and more mental health practitioners are trained using Westernized methods and conceptions. Mental healthcare practitioners, services, and policies will increasingly face normative issues as cultures all over the world have both their mental lives shaped (if not distorted) and their traditional ways of coping, influenced by the expansion of DSM taxonomy and nosology, as well as the conceptual assumptions and clinical data that comes with it (Murphy, 2015, p. 102, 103). Assessing whether someone’s life is not going well, as an inherent part of psychiatric practice, is always going to be a lot harder, and shakier, when it comes to subjects from very different cultural situations, comparatively to making them within the scope of one’s own culture (Murphy, 2015, p. 104).

While it is erroneously assumed that the human norm resides within Westerners’ minds, when it comes to affective psychology, the opposite happens: Cultural dependence and variety are readily assumed and taken at face value, while claims on human emotions’ universality are rarely heard. Indeed, whereas cognitive systems like vision are regarded as culture-independent, emotions seem to be the opposite: nothing but culture (Murphy, 2015, p. 104, 105).

The question of normativism and insufficient culturally diverse data is hinted at in a recent critical piece by the American psychiatrist Allen Frances, chair of the task force that oversaw and revised the development of DSM-IV:

“DSM-5-TR now greatly compounds DSM-5's colossal error by adding an even bigger one of its own—turning prolonged grief into an official mental disorder (…) ‘Prolonged’ is defined as just 1 year in adults and 6 months in kids. Why is this so destructive? There can never be a uniform expiration date on normal grief, and the DSM-5-TR should not feel empowered to impose one. People grieve in their own ways, for periods of time that vary widely depending on the person, the nature of the loss, and relevant cultural practices. (…) The decision to declare prolonged grief a psychiatric disorder was based on minimal research by just a few research teams; it has not been field tested in a wide array of practice settings (…) Psychiatric diagnosis for grievers is rarely necessary; only needed when patients meet full criteria for major depressive disorder and are experiencing clinically significant distress or impairment. (…) Experts always worry about missed patients and undertreatment, never about mislabelling ‘normal’ or overtreatment.” (Frances, 2022, March 23, emphasis added).

Following up on the research involved in the decision to formalize PGD, three of the DSM-5 Steering Committee leaders wrote the following:

“From November 2017 to November 2020, the Committee received 29 proposals. As of November 2020, these proposals had resulted in adoption of one new diagnostic category (Prolonged Grief Disorder) (…) This proposal was submitted by a major research group, which offered supporting post–DSM-5 data. Our Review Committee identified a need for additional data and for input from the other major research group studying adult PGD and from child PGD experts. (…) in June 2019 the APA sponsored a 1-day, in-person workshop that included the major research groups and an independent panel charged with making recommendations based on the workshop presentations and discussions. (…) The Steering Committee, the APA Board, and the APA Assembly approved the new PGD criteria. Although this process took more than 2.5 years from submission to final approval, the participants were satisfied that all voices had been heard and accommodated if possible, and the end result was well supported by the research literature” (Appelbaum et al., 2021, p. 1,348, 1,349, emphasis added).

Finally, a point I wish to address is the question of how culturally diverse is the evidence that was taken into consideration for the approval of PGD. I mean not to contest the existence of PGD in Western Societies, but are we to believe this is a universal condition? Are we to consider this is a cross-cultural disorder despite the fact that the concept was developed in Western countries, using Western concepts and Western psychometric constructs? While the actual empirical data that were analyzed by the DSM-5 Steering Committee are not divulged, one can go as far as to look at a surrogate set of data, as summarized in Table 2.

**Table 2:** Results from a surrogate search on the Medline database regarding prolonged grief between 2013 and November 2020.

Research team	Country of the research team	Country of the sample	Sample (*n*=)
Mauro et al. (2019)	USA	USA	261 (white (82%), non-Hispanic (87%), well educated)
Wenn et al. (2015) and Wenn et al. (2019)	Australia	Perth, Western Australia	22 (English speaking)
Bartl et al. (2018)	Germany	Germany	51 (fluent German language skills and literacy necessary)
Bryant et al. (2014, 2017)	Australia and Israel (1)	New South Wales, Australia	80 (13 years of educational level, on average; English speaking necessary)
Spuij et al. (2013) and Spuij et al. (2015)	Netherlands	Utrecht, Netherlands	10 (All 10 were Caucasian)
Skritskaya et al. (2020)	USA and Germany (1)	USA (Boston, New York, Pittsburgh, and San Diego)	394 (recruited from academic medical centers, predominantly Caucasian (*n* = 325) and highly educated sample)
Rosner et al. (2014, 2015)	Germany	Germany	51 (fluent German language skills and literacy)
Lichtenthal et al. (2019)	USA and Australia (1)	USA	8 (non-Hispanic white (88%), college educated (76%), employed (75%), Catholic (88%), vs. 1 Jewish participant; had to be English speaking)
Buck et al. (2020)	USA	USA	54 (93% white).
Kersting et al. (2013)	Germany	Münster, Germany	228 (participants were recruited through a website, 17 years of education on average)
Hall et al. (2014)	USA	USA (San Diego, Boston, New York, and Pittsburgh)	335 (predominantly white (85%), 87.46% had over 12 years of education)
Litz et al. (2014)	USA	USA	84 (57 female, 83 Caucasian, 77 went to college)
Supiano and Luptak (2014)	USA	USA	26 (mean age of 67 years, 20 female, 34 white)
Simon et al. (2020)	USA	USA	194 veterans or service members with combat-related PTSD ± diagnosis of complicated grief (113 white (vs. 64 Black, 33 Other), 23 Hispanic, 73 with high school or less).
Eisma et al. (2015)	Netherlands	Netherlands	47 pax with elevated levels of complicated grief, recruited via online questionnaire (28 with higher education).
Papa et al. (2013)	USA	USA	25 participants (convenience sample recruited via advertisements; white (vs. 1 Native American, 1 Latino) and most from lower/lower-middle class).
Brodbeck et al. (2017, 2019)	Switzerland	Switzerland	110 participants, but only 25 bereaved (mainly recruited by newspaper articles, 77 had attended college, 80 Swiss vs. 24 from German-speaking countries and 6 originally from other countries)
Sandler et al. (2016)	USA	USA	131 parents +206 offspring (Ethnicity of the parents was 67.89% non-Hispanic white, 10% Hispanic, 6% African American, 1.5% Native American, 1.5% Asian American or Pacific Islanders, and 9% other or unknown; median family income at pretest was in the range of $30,001 to $35,000).
Ghesquiere (2013)	USA	New York, USA	8 complicated grief-positive older adults (recruited via radio and newspaper advertisement and from referrals from other mental health professionals; 6 white, 6 completed college).
Saindon et al. (2014)	USA	USA	51 (37 European-American (72.5%) vs. 8 Hispanic, 4 African-American, and 2 “Other”; 24 attended college and 7 graduate school)
Kissane et al. (2016)	USA and Australia	USA	620 (Ethnicity 88.5% non-Hispanic and 11% Hispanic; Race 81.8% white, 9.8% Black, 3.2% Asian, 1.8% Other).
Duberstein et al. (2019)	USA	USA	55 bereaved participants who completed post-mortem assessments (75% with education beyond high school).
Dorsey et al. (2020)	USA, Kenya, and Tanzania	Kenya and Tanzania (both urban and rural)	640 children (320 girls and 320 boys; 630 still in school)

This query was undertaken using the terms “prolonged” and “grief,” the filters “Clinical Trial” and “Randomized Controlled Trial,” as well as restricting the publication date to the post-DSM-5 period mentioned in Appelbaum’s paper (between 2013 and November of 2020). Thirty-three results emerged, and 28 were condensed in the present table. Five articles were excluded on account of pertaining study protocols without accompanying data or the data-pertaining paper (*n* = 4) or because they did not measure grief (*n* = 1). This search was undertaken on 20 July 2022.

From this surrogate search, it is strikingly evident that there is a clear predominance of Western, Educated, Industrialized, Rich, and Democratic subjects among the sample-based empirical data on prolonged grief. While this observation is not absolute, one can wonder to what extent is PGD not a Western Cultural Syndrome. Why presume it can be found in other cultures if very little non-Western empirical data was taken into consideration? As we have seen earlier, loss responses are extraordinarily culture-relative.

Although DSM-5-TR makes several attempts to safeguard cultural differences along its main body of text, including in PGD via criterion E [“The nature, duration, and severity of the bereavement reaction must clearly exceed expected social, cultural, or religious norms for the individual’s culture and context” American Psychiatric Association (APA), 2022a, p. 324], and a paragraph on culture-related diagnostic issues [American Psychiatric Association (APA), 2022b, p. 326], this approach feels insufficient to safeguard non-Western bereaved subjects from being regarded as disordered, because it outright assumes the existence and validity of this diagnosis outside the dozen countries and cultures where it was studied, hence remaining heavily Western-born. Even though the DSM warns the clinician to be somewhat culture-relative, it does not warn of its normativism and possible culture relativity shortcomings, leaving clinicians to use their best clinical judgment on a bereaved person from a potentially wholly different cultural background.

Coming back to Murphy:

“DSM-5, despite its notionally greater openness to medical anthropology, retains many of the bad habits of earlier editions. In particular, it has not fully broken free of the idea that Western psychiatric categories represent normal deviance, and non-Western ones represent deviant deviance. (…) It is entirely reasonable to suggest that many disorders are experienced by Westerners in ways that reflect a distinctive cultural heritage that the rest of the world does not share, so that even if there are genuinely universal conditions, Westerners in fact may be the ones with the culture-bound syndrome.” (Murphy, 2015, p. 108).

Doubling down on Murphy’s appeal, this article tries to call on fellow mental health practitioners to be exceptionally careful, and even skeptical, of considering a PGD diagnosis for a non-*WEIRD* subject.

### 3.2 Future steps in the cross-cultural study of disordered grief

Understanding grief through a transcultural lens presents several challenges, such as the difficulty (but not impossibility) of suspending one’s own cultural frameworks. This last section aims to offer insights into augmenting transcultural perspectives on grief, anticipating potential counterarguments, and emphasizing the undeniable importance of comprehending the multifaceted nature of grief.

It is crucial to incorporate a broader array of voices, from various cultural backgrounds, on the empirical and conceptual substratum that leads psychiatry to contemplate disordered forms of grief within its nosology. In other words, the inclusion of diverse perspectives is necessary to address this conceptual difficulty in a pluralistic setting and manner. This includes not only scholars from the field of psychiatry but also researchers from social sciences (sociology, anthropology, and ethnography, to name a few), mourners, and cultural stakeholders involved in mental health assistance. Second, collaborative interdisciplinary research can provide a more comprehensive understanding of how grief manifests across different cultures and help counter Western-centric biases, particularly given the above-mentioned misrepresentation in empirical science. Encouraging and funding cross-cultural collaborative research initiatives are thus imperative. By fostering partnerships between researchers from Western and non-Western cultures, studies can explore the nuances of grief experiences and the culturally shaped manifestations. This approach would contribute to a more inclusive and representative body of knowledge, as well as mitigate the risk of both oversimplification and overgeneralization. A third recommendation would be to place the burden of proof, concerning transcultural validity, on the proposing nosology enterprise, namely, by making clearer this limitation, to prevent overgeneralization without hindering ongoing research. Finally, and on a more individual line for clinicians, Tantam’s recipe may be of use:

“Travel and wide reading are long prescriptions for developing knowledge and sympathy for other peoples and other cultures. (…) Travel, with its own small attendant desperations, and reading, with its opportunity to identify with heroes and heroines stricken by circumstance, might in lesser ways also increase our capacity to empathize. Both travel and reading can certainly open our eyes to other values, and other world views.” (Tantam, 2007, p. 385, 386).

Opponents of this project may argue that infusing more cultural relativism into the study of disordered grief, particularly PGD, would lead to a situation where there would be no universal standards for evaluating and managing grief. Yet, this argument presupposes, once more, that current practice is somehow inherently better and legitimate, despite the previously appointed lack of representativeness and validity. However, the potential to over- or underdiagnose (and treat) mourners from/in different cultural backgrounds, from those which its imperatives stem from, poses a sure wrong by any standards. A more fine-tuned and culturally relative diagnostic and, if necessary, therapeutic approach is ethically justifiable. It is essential to emphasize that transcultural understanding does not negate the existence of common, or even universal, aspects of grief (as was previously discussed with the biological design of loss responses) but rather seeks to elucidate those cultural factors that color, modulate, and add breadth to this complex human experience—foremostly, when does it warrant a mental healthcare approach. Second, some critics, persuaded by essentialist views, may argue that grief is a universal experience that transcends cultural differences, hence cultural relativism being dispensable. However, just because grief may have universal aspects does not mean it is universally the same. Among other things, grief is interpersonal, contextual, and historical, and as we have previously illustrated, culture contributes to content despite its biological design. Transcultural understanding enhances rather than diminishes the universality of grief by acknowledging the diverse ways it is expressed and coped with globally.

The undeniable cultural embeddedness of grief becomes apparent when examining how different societies ritualize mourning, commemorate the deceased, and conceptualize both the afterlife and life after the loss (see section 3.1.2.). Indian widows living in Fiji are expected to cease social participation and any contact with men (except for a few relatives), as well as to devote themselves to their children and widowhood. Consequently, depression frequently ensues (Tseng, WS, 2007, p. 105). If one were to follow criterion E of DSM-5-TR criteria (see Table 1), or the analogous ICD-11 constraint of “clearly [exceeding] expected social, cultural or religious norms for the individual’s culture and context.” (World Health Organization (WHO), 2022), then probably no PGD diagnosis could be made in this population, but if one were to overlook those constraints, then probably a great number of them would be diagnosable. Given these cultural contours, the question then becomes of how one might identify or even individualize a situation of prolonged grief in such a population. Again, are we even certain it exists naturally everywhere? Inversely, one could similarly be made to wonder whether PGD would develop at all among Samoan widows(ers), given how, in their culture, death is seen as a natural event in life, and ample family and community support is deployed to the relatives following the loss (Ablon, 1971), possibly curbing the development of PGD. By studying these cultural nuances, psychiatry enriches its understanding of grief, emphasizing that what may seem pathological in one culture could be normative in another. In a globalized world where migration, resettlement, and forced displacement keep taking place, and Western psychiatry has become an export to both the global South and Eastern countries, this latitude matters plenty, especially if we consider how the worsening climate crisis might promote further migratory fluxes.

To reinforce the transcultural validity of disordered grief, the inclusion of perspectives from non-Western thinkers is paramount. This may be achieved through the work of several scholars (see below), who have provided valuable insights into the cultural dimensions of mental health, challenging Western-centric perspectives and contributing to a more inclusive discourse on grief; but also, through active involvement of indigenous populations, presenting them the opportunity to directly influence policies and practices regarding grief support, under the appropriate cultural frameworks. The Māori-lead *He Oranga Ngakau* report offers a fine example of how a care approach may be developed and spear-headed by those entrenched in the culture it is designed to serve (Pihama et al., 2020).

Considering the limited depth and illustration offered, this study may be aptly complemented by further explorations into the wealth of case studies and ethnographic research available in the literature, concerning grief and coping mechanisms, to offer a more nuanced understanding of how grief is navigated in various cultural contexts. Views from authors who have addressed transcultural aspects of grief may be useful, such as Donald P. Irish (see Irish et al., 1993), Colin Murray Parkes (see Murray Parkes, 1998; Parkes et al., 2015), or Joan Ablon (see Ablon, 1971).

## 4 Conclusion

Much of today’s discourse on prolonged grief focuses on the extremes of the spectrum, relinquishing a healthier and more productive discussion on the borderland and its presupposed transcultural validity. While this bicephalic approach (and further entrenchment) rages on, people who may actually be suffering from pathological forms of grief, or are at a greater risk of developing them, continue to have their mental health needs overlooked by themselves, their close ones, and/or even their health practitioners. This entrenchment hinders research studies and enables the undue overspread of this Western category into other cultures.

It is my hope that this article incites a more profound reflection and discussion on the limits of normal grief, including, but not exclusively, from our *WEIRD* point of view.

## Author contributions

AG conceived and designed the study, acquired, analyzed, and interpreted the data for the study, drafted the study and revised it critically for important intellectual content, provided approval for publication of the content, and agreed to be accountable for all aspects of the study in ensuring that questions related to the accuracy or integrity of any part of the study are appropriately investigated and resolved.
